# Acceptability of community quarantine in contexts of communicable disease epidemics: perspectives of literate lay people living in Conakry, Guinea

**DOI:** 10.1017/S0950268819001419

**Published:** 2019-08-01

**Authors:** Lonzozou Kpanake, Jean-Pierre Leno, Paul Clay Sorum, Etienne Mullet

**Affiliations:** 1 University of Québec – Teluq, Montréal, Canada; 2 University of Conakry, Conakry, Guinea; 3 Albany Medical College, Albany, New York, USA; 4 Institute of Advanced Studies (EPHE), Paris, France

**Keywords:** Acceptability, communicable diseases, epidemic, perception, quarantine

## Abstract

During the 2014–2016 Ebola epidemic in West Africa, some communities reacted hostilely to the implementation of quarantine measures. This study's aim was to examine the views of lay people in Guinea on the acceptability of community quarantine. From June to August 2016, 302 adults indicated the acceptability of quarantine in 36 scenarios varying as a function of four factors: the infectious disease's level of contagiousness, its level of lethality, the number of cases in the community and whether persons in quarantine are provided with support services. Five clusters were identified: (1) for 18% of the participants, quarantine is never acceptable; (2) 16% considered, in contrast, that quarantine is always acceptable; (3) for 14%, it depends on the disease's level of contagiousness and lethality; (4) 36% based their judgement not only on the levels of contagiousness and lethality, but also on whether those in quarantine are provided with support services; and (5) 16% had no opinion. Interventions to increase voluntary compliance with community quarantine in Guinea must not be ‘one size fits all’, but must be multifaceted and tailored in design and implementation to match the diversity of people's concerns and needs.

## Introduction

Community quarantine is ‘the compulsory physical separation, including restriction of movement, of populations or groups of healthy people who have been exposed to a contagious disease. This may include efforts to segregate these persons within specified geographic areas’ [1]. It is one of the oldest tools to control communicable disease epidemics [2, 3]. During the 2014–2016 Ebola epidemic in West Africa, with no effective vaccines or treatment, the most affected West African countries – Liberia, Sierra Leone and Guinea – resorted to community quarantine as the mainstay means to control the spread of Ebola [3, 4]. Such measures were, however, opposed by some communities who reacted hostilely [5–7], which in turn prompted local authorities to resort to violence [6–8]. In Liberia, for instance, soldiers and armed police were deployed to quell a community opposed to quarantine, with one boy shot dead and many injured [7].

The refusal of several West African communities to comply with quarantine measures raises an important question about the acceptability of such a strategy as a means to control communicable disease epidemics. Whether community quarantine is acceptable is controversial. Broadly speaking, the debate on the issue can be categorised into three perspectives:

*Protect the health of the community*: Supporters of this view consider community quarantine as the only way to contain the spread of a highly lethal communicable disease of which very little is known [2, 9]. Its justification stems from a general moral obligation to prevent harm to others if this can be done [10]. Most countries have laws that permit the issuing of quarantine orders. Such measures are credited with slowing the rate of spread and minimizing the rate of death during the 2003 pandemic of severe acute respiratory syndrome (SARS) [11] and the 1918–1919 Spanish flu pandemic [12].

*‘Do no harm’ to individuals*: Some commentators argue that the use of community quarantine was in fact neither effective nor efficient in controlling the spread of infections such as SARS [13] or Ebola [14, 15] and that it should not be considered ‘a primary public health strategy in most imaginable circumstances’ [16]. Others object that it is unfair to justify the use of quarantine solely on the basis of public health interests. They hold that individual persons should be valued as ends in themselves and should never be used as a means for public health ends [17]. Bensimon and Upshur argue that in the world in which quarantine was first conceived and enforced, individuals had an obligation to respect restrictive measures, but that in today's fundamentally different democratic societies, individuals have the right to protest such deprivation of liberty [2]. Furthermore, while the effectiveness of quarantine remains uncertain, numerous post-epidemic investigations have shown harmful consequences in those quarantined: symptoms of depression and post-traumatic stress disorder [18], experiences of stigma [19], social isolation [20] and loss of household income [21].

*Depends on circumstances*: For many commentators, even though community quarantine is a curtailment of civil liberties, it can be justified if several criteria can be met. According to Kass, for instance, ‘programs that are coercive should be implemented only in the face of clear public health need and good data demonstrating effectiveness’ [22]. Upshur proposes a four-principle public health ethics framework that can justify an implementation of community quarantine: ‘First, […] this infection should be spread from person to person […]; secondly, public health authorities should use the least restrictive measures proportional to the goal of achieving disease control […]; thirdly, if society asks individuals to curtail their liberties for the good of others, society has a reciprocal obligation to assist them in the discharge of their obligations […]; and finally, public health authorities have an obligation to communicate clearly the justification for their actions and allow for a process of appeal’ [23].

Despite the long and controversial history of the use of community quarantine, empirical data on how the public see this issue are lacking. This knowledge is, however, important as it can help public health policy-makers to tailor interventions and education that will take into account people's concerns and needs and that will thereby increase voluntary compliance with community quarantine if and when needed in the future.

The limited studies about public attitudes towards quarantine measures have been conducted exclusively in high-income countries – e.g. Canada, Hong Kong, Singapore and Taiwan – and indicated strong public support for the use of quarantine in contexts of infectious disease epidemics [11, 24]. Public support for the use of quarantine in those countries contrasts with the attitudes of non-compliance observed in West African contexts and, therefore, intensifies the need to understand how quarantine is perceived in West Africa.

The purpose of the present study was to examine the views of lay people in Guinea on the acceptability of community quarantine in different circumstances varying as a function of contagiousness of the disease, lethality of the contagious disease, number of cases and humanitarian conditions. Its purpose was not to study the participants' views of the quarantine measures implemented in Guinea during the 2014–2016 Ebola epidemic. The study's purpose was also not epidemiological, i.e. it did not try to provide accurate estimates of the percentages of lay people in Guinea who would see quarantine as acceptable or not and for what reasons. Instead, its purpose was psychological – to map the various positions taken by different groups of people. This knowledge could in turn provide insights about the design and implementation of tailored interventions that could increase voluntary compliance with community quarantine.

## Method

### Study design

The present study used a vignette methodology. A vignette is a hypothetical situation to which research participants respond thereby revealing their views or values. We presented participants with vignettes depicting the implementation of quarantine measure in a community affected by a communicable disease epidemic, and instructed them to indicate the extent to which such a measure would be acceptable to them. The vignettes were composed of combinations of different levels of four factors influencing perceptions of quarantine, as suggested by previous studies [3, 11, 14, 24]: (a) the infectious disease's level of lethality; (b) the disease's degree of contagiousness; (c) number of cases identified in the community; and (d) whether persons in quarantine are provided with basic support services. The validity of vignette methodology for empirical investigation of controversial and ethically challenging issues was supported by Ulrich and Ratcliffe [25], by Mah *et al*. [26] and by Wainwright *et al*. [27]. According to Ulrich and Ratcliffe [25], the vignette technique makes it possible to assess how cues are weighted, how they are combined and how different groups of participants differ in weighting and combining. One condition for examining the processes of weighting and combining, independently of other processes, is that each participant has the same information presented in the same way. The vignette technique has already been used in several studies on ethically challenging issues in Africa, such as resorting to criminal law as a means to control HIV epidemic [28], allocation of scarce medical resources [29] and physicians' duty to provide care during Ebola epidemics [30].

### Study setting

The republic of Guinea is a West African country with a population of 12 609 000 [31]. It was one of the countries most affected by the 2014–2016 Ebola epidemic, with 3811 cases and 2543 deaths [32]. To interrupt transmission chains of the virus, the Guinean government and a variety of international organisations implemented various containment strategies, including community mobilisation programmes, health promotion and the reinforcement of standard precautions, safe burials of Ebola victims, isolation and management of confirmed Ebola cases in designated healthcare facilities with maximal biosafety procedures, isolation of suspected cases, Ebola case finding – through active surveillance, follow-up of rumours and contact tracing – mandated hand washing at entry of public places, abolition of hand shaking practices, mandated fever screening before entry of public buildings, closures of public places, curfews, closing of borders, social distancing measures and community quarantine [3, 14, 33, 34].

On 1 August 2014, local authorities established a quarantine zone of 20 000 km^2^, erecting a barrier to isolate the major Ebola epicentre, with strict enforcement by the military [35]. In addition, individual quarantine measures were applied to over 54 500 people throughout the country who had been potentially exposed to Ebola cases [33]. Quarantined people were monitored for the development of symptoms of Ebola to ensure appropriate isolation and treatment. Many of those people lost their livelihoods, lacked basic commodities, but did not receive the support services – food and water – promised by the Government [34]. Numerous hostile reactions to quarantine measures were reported, including vandalism, death threats and physical aggression towards medical teams monitoring quarantined persons [34–37].

### Participants

Data collection for this study started after the World Health Organization declared the end to the Ebola epidemic in the country on 1 June 2016. From June to August 2016, nine research assistants, trained in the methodology used for the present study – Anderson's functional theory of cognition [38] – approached persons walking along the main sidewalks of the five districts of Conakry: Kaloum, Dixinn, Ratoma, Matam and Matoto. The researchers predefined strata (e.g. gender, age group and religious background) within the literate people in Guinea and then conveniently selected participants belonging to each strata. Given that the research language was French, the official language in Guinea, only people capable of understanding and reading French were selected in order to prevent any influence of translation by the researchers on participants' responses. Three hundred and fifty persons were selected and received full explanations regarding the study and the procedure. Those who agreed to participate provided written informed consent. They received no incentive.

### Material

The material consisted of 36 cards containing a story of a few lines, a question and a response scale. The stories depicted the imposition of mass quarantine measures in a community affected by a communicable disease epidemic. They were designed according to a four within-subject orthogonal design (3 × 2 × 3 × 2):

1. The infectious disease's level of lethality:
  - very high: fatality rate of about 90%;
  - high: fatality rate of about 50%; or
  - relatively low: fatality rate of about 10%
2. The disease's degree of contagiousness:
  - highly contagious, or
  - moderately contagious
3. Number of cases identified in the community:
  - high: 100 cases in a community of 2000 inhabitants;
  - intermediate: 50 cases in a community of 2000 inhabitants; or
  - relatively low: five cases in a community of 2000 inhabitants
4. Whether persons in quarantine are provided with basic support services:
  - they are provided with food, water and medical supplies; or
  - no support is provided

Under each story were a question – ‘To what degree does the implementation of quarantine measures in this case seem acceptable to you?’ – and an 11-point response scale with a left-hand anchor of ‘Not at all acceptable’ (0) and a right-hand anchor of ‘Completely acceptable’ (10). The cards were arranged in random order for each participant. Two examples of scenarios are given in the online Supplementary Appendix A.

### Procedure

Participants were given a choice of test site: either right away in a quiet classroom in a local school, at a 3 min walk, or later at their private homes. Seventy-eight per cent chose to be tested at the school.

All participants were provided with a standardised definition of *community quarantine* [‘The compulsory physical separation, including restriction of movement, of populations or groups of healthy people who have potentially been exposed to a contagious disease’]. Researchers explained to each participant what was expected: that he or she was to read 36 stories in which a community affected by a communicable disease epidemic is put in quarantine, and was to indicate, in each case, the degree of acceptability of such a measure. Participants were tested individually, according to the procedure recommended by Anderson [38]. They made ratings themselves, at their own pace, and the researchers routinely made certain that each participant, regardless of age or educational level, was able to grasp all the necessary information before making a rating. The participants took 30–45 min to complete the ratings.

Ethics approval for the study was obtained from the Guinean National Review Board for Health Research, the Guinean National Review Board for Research on Ebola and the Institutional Review Board of the University of Quebec (Teluq). Full anonymity was provided to all participants.

### Statistical analyses

For each of the 36 scenarios, the mark on the response scale was converted into a numerical value ranging from 0 to 10. We conducted a cluster analysis on the raw data, using the *K*-means method, as recommended by Hofmans and Mullet [39]. Clustering procedures enable the grouping of study participants according to the way they weight and combine information when forming acceptability judgements [39]. Numerous previous studies have used these procedures in order to identify subgroup differences in acceptability judgements [28–30, 40, 41]. A five-cluster solution was retained based on the technique advocated by Schepers and Hofmans [42]. An overall analysis of variance (ANOVA) was then conducted on the raw data with a design of Cluster × Contagiousness × Lethality × Support × Incidence, 5 × 2 × 3 × 2 × 3. The Cluster effect was significant, and three of the four two-way interactions involving the clusters factor were significant at the 0.001 level. As a result, five separate ANOVAs were conducted on the data of each cluster, using a design of Contagiousness × Lethality × Support × Incidence, 2 × 3 × 2 × 3. Owing to the multiple comparisons made, the significance threshold was set at 0.001. Finally, we performed probability comparison tests to examine the effects of demographic characteristics.

## Results

Of the 350 persons contacted, 302 (165 women and 137 men) agreed to participate. Their ages ranged from 18 to 67 years (mean = 27.18 years, standard deviation: 4.21). Forty per cent self-identified as Muslim, 39% as Christian, 16% as Animists and 5% as atheists. They had completed at least an elementary-school education. More detailed demographic information is shown in Table 1. Rates of literacy and elementary school attendance among the participants were higher than those of the general population of Guinea (which were in 2013, 25.3% and 83.5%) [43]. No reliable statistics are available for the other demographic characteristics considered in the study.

**Table 1.** Demographic characteristics of the whole sample and distribution of participants in the five clusters

	Cluster					
Characteristic	Never acceptable	Support, contagiousness and lethality	Contagiousness and lethality	Always acceptable	No opinion	Total
Gender						
Male	15 (11)a	49 (36)	17 (12)	29 (21)^a^	27 (20)	137
Female	40 (24)a	59 (36)	24 (15)	20 (12)^a^	22 (13)	165
Age						
18–20 Years	14 (21)a	13 (20)a,b	2 (3)a,b	21 (31)a,b,c	17 (25)a,b	67
21–30 Years	22 (27)b	16 (20)c	8 (10)c	10 (12)a	25 (31)c	81
31–40 Years	13 (18)	31 (43)a,c	19 (26)^a,c^	4 (6)b	5 (7)^a,c^	72
40 + Years	6 (7)a,b	48 (59)b,c	12 (15)b	14 (17)c	2 (2)b,c	82
Religion						
Christian	6 (5)a,b	65 (53)a,b,c	15 (12)a	18 (15)	18 (15)	118
Muslim	31 (26)a	31 (26)^a^	10 (9)b	21 (18)	25 (21)	122
Animist	16 (34)b	11 (23)b	6 (13)c	8 (17)	6 (13)	47
Atheist	2 (13)	1 (7)c	10 (67)^a,b,c^	2 (13)	0 (0)	15
Educational level						
Elementary	27 (39)^a,b,c^	19 (27)^a^	8 (11)^a^	8 (11)	8 (11)^a^	70
Middle	16 (19)^a,b,c^	29 (35)^b^	20 (24)^a,b,c^	11 (13)	7 (9)^b^	83
High	10 (11)^b^	30 (32)^c^	8 (8)^b^	21 (22)	26 (27)^a,b^	95
College	2 (4)^a,c^	30 (56)^a,b,c^	5 (9)^c^	9 (17)	8 (15)	54
Total	55 (18)	108 (36)	41 (14)	49 (16)	49 (16)	302

The figures in parentheses are percentages.

Figures with the same subscript in each column are significantly different, *P* < 0.05.

For example, (A) in the second column, ^a^significantly higher number of women in the *Never Acceptable* cluster than of men; (B) in the fourth column, ^b^significantly higher number of atheists in the *Contagiousness and Lethality* cluster than of Muslims; and (C) in the fifth column, ^c^significantly higher number of younger participants (lesser than 20 years) in the *Always Acceptable* cluster than of older participants (above 40 years).

**Fig. 1.**
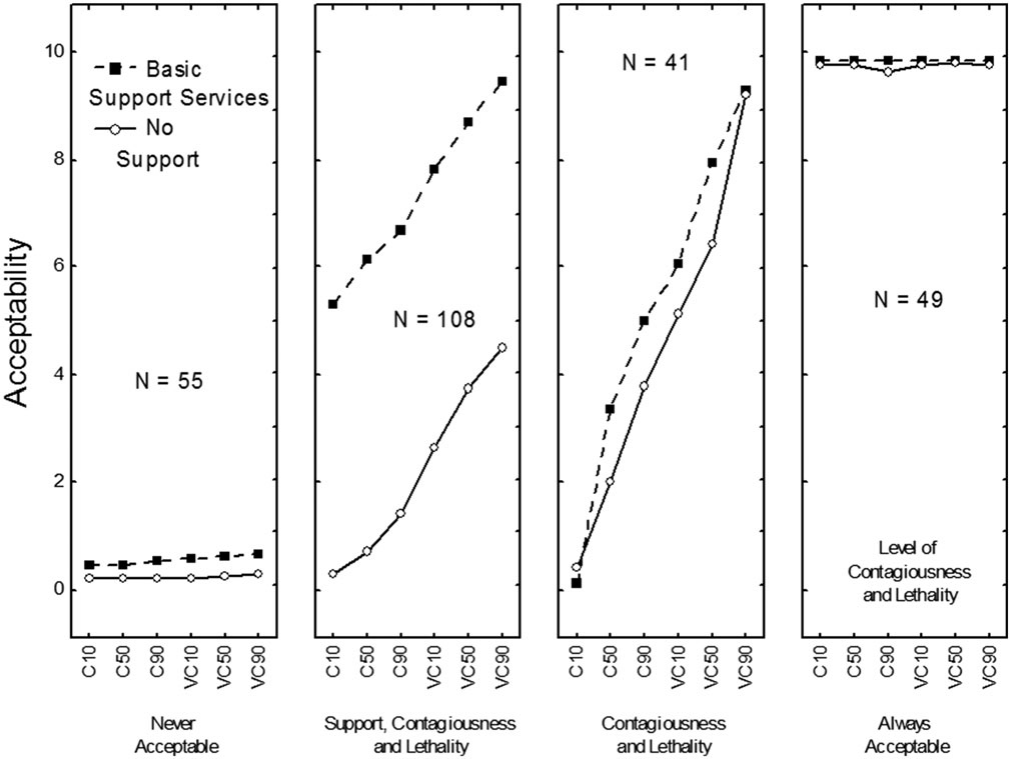
Patterns of results corresponding to four of the five clusters: “Never Acceptable”, “Depends on Support, Contagiousness, and Lethality”, “Depends on Contagiousness and Lethality”, and “Always Acceptable”. In each panel, 1) the judged acceptability of quarantine is on the *y*-axis; 2) the three levels of the disease’s level of lethality and the two levels of contagiousness are on *x*-axis; C10: The disease is moderately contagious and its fatality rate is about 10%; C50: The disease is moderately contagious and its fatality rate is about 50%; C90: The disease is moderately contagious and its fatality rate is about 90%; VC10: The disease is highly contagious and its fatality rate is about 10%; VC50: The disease is highly contagious and its fatality rate is about 50%; VC90: The disease is highly contagious and its fatality rate is about 90%; and 3)  the two curves correspond to whether support services were provided or not.

The patterns of data that correspond to four of the five clusters are shown in Figure 1, and the distribution of participants in each cluster is shown in Table 1. Mean ratings for each scenario, overall and for each cluster, are available from the corresponding author.

The first cluster (*N* = 55, 18% of the sample) was called *Never Acceptable* because the mean rating was 0.30; that is, extremely close to zero. The impact on ratings of the factors involved in the scenarios was weak. Females (24%), younger people (21% of ages 18–20 and 27% of ages 21–30), Muslims (26%), Animists (34%) and participants with an elementary-school education (39%) were significantly more likely to belong to this cluster than other participants.

The second cluster (*N* = 49, 16% of the sample) was called *Always Acceptable* because the mean rating was 9.80; that is, extremely close to the end of the acceptability scale. The impact on ratings of the factors involved in the scenarios was not significant. Males (21%) and the youngest participants, aged 18–20 (31%), were significantly more likely to belong to this cluster than other participants.

The third cluster (*N* = 41, 14% of the sample) was called *Depends on Contagiousness and Lethality* because the impact of only these two factors was strong. Ratings were higher when the level of contagiousness was high (*M* = 7.35) than when it was moderate (*M* = 2.44), *F*(1,38) = 1666.73, *P* < 0.001, *η*^2^_p_ = 0.98, and when the level of lethality was very high (*M* = 6.83) than when it was high (*M* = 4.93) or low (*M* = 2.93), *F*(2,76) = 281.91, *P* < 0.001, *η*^2^_p_ = 0.88. The impacts of support services and the number of cases of infection were weaker; mean ratings ranged from 4.50 (no support) to 5.29 (support provided), and from 4.69 (five infection cases) to 5.15 (100 infection cases). Participants aged 31–40 years (26%), atheists (67%) and participants with middle-school education (24%) were significantly more likely to belong to this cluster than other participants. Overall, in this cluster, acceptability ratings were higher than 7 (out of 10) in only nine cases.

The fourth cluster (*N* = 108, 36% of the sample) was called *Depends on Support, Contagiousness and Lethality* because the impact of all three factors on acceptability judgements was strong. Ratings were considerably higher when support services were provided (*M* = 7.35) than when they were not (*M* = 2.20), *F*(1,105) = 816.56, *P* < 0.001, *η*^2^_p_ = 0.89. Ratings were higher when the level of contagiousness was high (*M* = 6.14) than when it was moderate (*M* = 3.42), *F*(1,105) = 471.84, *P* < 0.001, *η*^2^_p_ = 0.82. Finally, ratings were higher when the disease's level of lethality was very high (*M* = 5.50) than when it was high (*M* = 4.83) or low (*M* = 4.01), *F*(2,210) = 173.32, *P* < 0.001, *η*^2^_p_ = 0.62. The impact of the number of cases of infection was weak; mean ratings ranged from 4.61 (five cases) to 4.95 (100 cases). Participants older than 31 years (51%), Christians (53%) and participants with a college degree (56%) were significantly more likely to be members of this cluster than other participants. Overall, in this cluster, acceptability ratings were higher than 7 in only nine cases; six of these were the same cases as those rated over 7 in the third cluster.

The fifth cluster (*N* = 49, 16%) was called *No Opinion*. The mean rating was located near the middle of the response scale (*M* = 5.37), and the impact on ratings of the factors involved in the scenarios was not significant. Younger participants (25% of ages 18–20 and 31% of ages 21–30) and participants with a high-school education were significantly more likely to belong to this cluster than other participants.

Four additional ANOVAs were conducted with Gender, Age, Religion and Educational level as between-subject factors. Men's ratings (*M* = 5.49) were higher than women's (*M* = 4.48), *F*(1,292) = 9.96, *P* < 0.002, *η*^2^_p_ = 0.03. The main effect of age was not significant, but the effects of contagiousness and lethality were stronger among older participants than among younger ones, *F*(3,290) = 29.51, *P* < 0.001, *η*^2^_p_ = 0.23 and *F*(6,580) = 19.85, *P* < 0.001, *η*^2^_p_ = 0.17, respectively. The main effect of religious affiliation was not significant, but the effects of contagiousness and lethality were stronger among atheists than among other groups, *F*(3,290) = 11.45, *P* < 0.001, *η*^2^_p_ = 0.11 and *F*(6,580) = 13.52, *P* < 0.001, *η*^2^_p_ = 0.12, respectively. Finally, the main effect of education was significant. Ratings from participants with elementary education (*M* = 3.77) or middle-school education (*M* = 4.56) were lower than ratings from participants with higher degrees (*M* = 5.61), *F*(1,292) = 7.15, *P* < 0.001, *η*^2^_p_ = 0.07.

## Discussion

The issue of how to make community quarantine acceptable to the public is important for planning for and responding to future communicable disease epidemics in Africa. The present study examined the views of lay people in Guinea on the acceptability of community quarantine and found different positions, consistent with similar diversity among commentators in the literature [17, 22, 23]: (1) for 18% of the participants, quarantine measures are never acceptable, even when the disease is highly lethal and contagious, its incidence is high, and support services are provided; (2) 16% considered, in contrast, that resorting to quarantine as a means to control the spread of an infectious disease is always acceptable; (3) for 14%, it depends on the disease's level of contagiousness and lethality; and (4) 36% based their judgement not only on the levels of contagiousness and lethality, but also on whether those in quarantine are provided with support services. The other participants (16%) did not take a clear position on the issue, irrespective of circumstances. This spread of views echoes the findings of previous studies on attitudes towards the use of community quarantine in public health emergencies in Hong Kong, Singapore, Taiwan and the USA [24] and in Canada [11]; and reflects the controversial debate on the issue [2, 17].

Most of the participants (66%) understood that, even though community quarantine is a curtailment of individual liberties, it can be justified if three criteria are met: (1) infected persons are contagious; (2) the disease is lethal in most cases; and (3) those in quarantine are provided, at the least, with basic needs (food, water and medical care). These findings are consistent with those of Tracy *et al*. [11] that the vast majority of lay people in Canada ‘were in favour of safeguards against unwarranted and/or inappropriate use of quarantine’ and ‘strongly support that those affected with quarantine should be provided with social support’. These findings are also consistent with Upshur's [23] ethical framework for the justification of quarantine measures. Accordingly, to engender acceptance and strong public support for community quarantine in future communicable disease epidemics, health authorities must demonstrate to the public that infected persons are really contagious (even before they are sick enough to seek medical care), that consequences of the disease may be severe, and that those in quarantine will be provided with support services.

The importance of the first criterion was demonstrated in the 2014–2016 Ebola epidemic in Africa. Many researchers questioned the appropriateness of the use of quarantine during that epidemic given that scientific evidence indicates that those infected with Ebola are not contagious until they display the symptoms of the illness [14, 15]. Reflecting on this controversial issue, Calain and Poncin charged that the aim of the quarantine was to control the movement not of people who had been exposed to Ebola but of people whom the authorities did not trust to report their symptoms [14]. The use of quarantine in this case was perceived by communities as an arbitrary enforcement [14]. The importance of the third criterion – providing those in quarantine with basic needs (e.g. food, water, medical supplies), lost income and means of communication with their relatives living outside the quarantine zone – was demonstrated by the role of support services in the high voluntary compliance with quarantine measures during the 2003 SARS epidemics in Taiwan, China [24] and Canada [44]. However, providing support services to quarantined communities may be challenging in low-resource countries in West Africa, where infrastructures are poor and health systems under-resourced. Indeed, during the 2014–2016 Ebola epidemic in West Africa, the unprecedented spread of the virus in Guinea, Liberia and Sierra-Leone has been linked to factors such as poor infrastructures, severe deficiencies in logistical and transport systems, and lack of investment in the purchase of needed supplies [45]. Thus, improvement in these factors would be clearly important to ensure the effectiveness of quarantine measures.

A group that opposed quarantine under all conditions was also found in South Korea in the context of the spread to South Korea in 2015 of the Middle East Respiratory Syndrome [46]. While the researchers explained this attitude in South Korea by the respondents' specific belief that community quarantine may increase, rather than decrease, the spread of infections, the opposition of some people in Guinea to quarantine during the 2014–2016 Ebola epidemic can be explained, as suggested by Calain and Poncin [14], by more general doubts about the effectiveness of and optimal use of social distancing measures. Indeed, contrary to many other public health interventions – e.g. vaccination, sanitation and hygiene interventions, and promotion of healthy lifestyle – the use of quarantine has generally lacked strong empirical validation of effectiveness [13–15]. Bensimon and Upshur recounted such concerns, noting that ‘invoking quarantine raised difficult questions about the justifiability of an intervention that may or may not be effective’ [2]. The opposition to quarantine can also be explained, as suggested by previous studies' findings in Guinea [36, 37], by a general distrust of public health measures in the country. Thus, interventions to increase the acceptability of quarantine in future epidemics in Guinea should (a) use sound scientific evidence of effectiveness in a well-planned public education programme and (b) contain strategies aimed at building public trust in the institutions involved with Guinean public health. One possible approach may be the inclusion in the development and implementation of quarantine measures of trusted and credible community figures such as spiritual and traditional leaders as well as political leaders. These efforts would be appropriate responses as well to the small but significant group (16%) who did not take a position on the issue as a result, possibly, of being reluctant to show their opposition to an official policy, of being unwilling to take a clear position on such a controversial issue, or of not understanding the task.

Finally, this study found an effect of socio-demographic variables – e.g. gender, age and religion – on the perception of quarantine. While these findings are consistent with those of previous studies [11, 24], it is not certain that beliefs and social attributes associated with socio-demographic variables underlay respondents' positions. This issue should be explored through in-depth individual interviews.

This study has limitations. First, it used a convenience sample of only moderate size, restricted to participants who lived in Conakry, the capital city, and who could read and understand French. Therefore, our findings are not likely to be representative of the whole lay people in Guinea, which is largely rural and mostly illiterate. Future studies should examine the views of those other segments of lay people in Guinea. Second, it asked participants about fictional vignettes, not about real quarantined communities. The use of vignettes, however, is useful – it permits statistical analyses to reveal how people weight and combine separate factors. Third, while the use of names of real places in Guinea in the vignettes facilitated the design of realistic stories, this might have introduced a bias in the answers if participants could associate different scenarios with places they knew personally. Fourth, the researcher did not ask further questions to the respondents to elucidate the reasons underlying their viewpoints. Future follow-up studies using qualitative methods are needed to understand the respondents' justifications.

Although this study's findings cannot be interpreted as representing the views of the whole lay people in Guinea, they provide some insights and are hypothesis generating. The views of lay people in Guinea cannot be ignored in planning for and responding to future communicable disease epidemics since their compliance is vital to ensure the effectiveness of quarantine. Interestingly, this study's findings showed that the vast majority of respondents do not reject quarantine in an absolute manner; that is, when they are aware of the contagiousness and severity of the disease, and those in quarantine are provided with adequate support services, they see quarantine as acceptable. The diversity of positions, however, strongly suggests that, when resorting to quarantine as a means to control communicable disease epidemics, no one single strategy to promote voluntary compliance will be appropriate.

## Supplementary material

For supplementary material accompanying this paper visit https://doi.org/10.1017/S0950268819001419. click here to view supplementary material
