# Supplementary material for: Acceptability of community quarantine in contexts of communicable disease epidemics: perspectives of literate lay people living in Conakry, Guinea
Source: Epidemiol Infect. 2019 Aug 1;147:e248. doi: 10.1017/S0950268819001419 (PMC6805739; doi:10.1017/S0950268819001419)
Supplement: Supplementary file 1 [file S0950268819001419sup001.docx]

**Journal name:** *Epidemiology and Infection*

**Title of the article:** Acceptability of Quarantine in Contexts of Communicable Disease Epidemics: Perspectives of Lay People in Guinea

**Names of the authors:** Lonzozou Kpanake, Jean-Pierre Leno, Paul Clay Sorum, Etienne Mullet

**Appendix A**

Two examples of scenario

I

In the community of Makoko (2000 inhabitants), 100 inhabitants are sick and the analyses have detected the presence of a virus that is still incompletely studied.

It is known that the disease is extremely contagious; simple bodily contact can be sufficient to transmit the disease.

The disease is one of most serious that is known. At present, almost all (9 out of 10) infected people die from it, even if treatment is started right away.

The health authorities of the country have decided to put this community in quarantine for 21 days. No one is to leave the zone of the community.

A service to supply food, water, and necessary products is planned. Moreover, a medical center is operating in the community with skilled doctors and nurses to provide treatment.

*To what degree does the implementation of quarantine measures in this case seem acceptable to you?*

Not at all acceptable o---o---o---o---o---o---o---o---o---o---o Completely acceptable

II

In the community of Hamdalaye (2000 inhabitants), 5 inhabitants are sick and the analyses have detected the presence of a virus that is still incompletely studied.

It is known that the disease is quite contagious ; simple bodily contact is not sufficient to transmit the disease but eating from the same plate or sharing the same bed is often enough for the disease to be transmitted.

The disease is quite serious. At present, 1 out of 10 infected people die of it, even if treatment is started right away.

The health authorities of the country have decided to put this community in quarantine for 21 days. No one is to leave the zone of the community.

No service to provide food, water, or necessary products has been planned. The community must feed itself from existing reserves. No medical assistance has been planned. A dispensary is operating in the community with one nurse; it can give only painkillers.

*To what degree does the implementation of quarantine measures in this case seem acceptable to you?*

Not at all acceptable o---o---o---o---o---o---o---o---o---o---o Completely acceptable
